# Remedies and Their Uses

**Published:** 1907-02-23

**Authors:** 


### Remedies and their Uses.

### ACETOZONE.

### Antiseptic, Germicide, for External and Internal Use.

This substance, introduced by Parke, Davis and Co., 111 Queen Victoria Street, London, is an organic peroxide, and is said to be one hundred times more powerful as a germicide than peroxide of hydrogen. As dispensed, it is a white powder containing 50 per cent. pure acetozone, which is chemically a peroxide of benzoyl and acetyl ($C_6H_5.CO.O.O.CO.CH_3$). This powder keeps well, but it must be protected in carefully stoppered bottles from heat, damp, and all contact with organic matter. It is soluble in water to the extent of 1 part in 1,000 to 1 in 10,000, according to temperature, the age of the solution, etc. It may also be dissolved in neutral petroleum oil, ether, or chloroform, and to a certain extent in alcohol. It should, however, never be kept in solution for any length of time, as it gradually decomposes in all solvents with the exception of neutral petroleum.

Physiologically, besides its antiseptic action, it is a diuretic, but is otherwise practically inert. It has been employed with much success in conditions for which peroxide of hydrogen has been recommended, and is said to be specially valuable as an intestinal antiseptic in typhoid fever. With regard to the best method of administering the drug, it appears certain that water as a solvent is preferable to other media, as it assists in the liberation of the nascent oxygen on which the action of acetozone depends. It appears, moreover, inadvisable to give acetozone in capsules, as the drug, when liberated in the stomach, may irritate the mucosa. The aqueous solution should be made by shaking up twenty grains in a quart of warm distilled water for five minutes. It should then stand for two hours, and the doses decanted off as required. If preferred, the doses may be flavoured with orange, ginger, lemon, or saccharin, or given in syrup or some effervescing water; but the stock solution must not be mixed with any foreign substance. In some cases it may be necessary to begin with a weaker solution and gradually to increase the strength. In a cold, dark place, such as a refrigerator, such a solution will keep for at least a fortnight, so that if much is required a gallon jar may be prepared at a time. A cellar may be used to store it in the absence of a refrigerator. Although distilled water is not absolutely necessary, it is better to use it if possible. In no case should a " hard " water be employed, as acetozone is easily decomposed by carbonates. It should not be given directly after food, as it will then be too rapidly decomposed in the stomach.

### Its Therapeutic Uses.

For external use the solution must be filtered and mixed with two or three parts of cooled boiled water. As a dusting powder acetozone may be mixed with 100 to 1,000 parts of boracic acid powder or talc. Ointments should be made with a paraffin basis only, and may be of .5 per cent. strength to begin with. If necessary, stronger preparations may subsequently be employed.

For use in a nebuliser, a special preparation is on the market, but a solution which will be effective may be prepared in the following manner: 1 part of acetozone is added to 100-500 parts neutral liquid paraffin previously warmed to 130° F. The mixture is well shaken and then filtered before it cools.

In typhoid fever the usual method af administering this drug as practised in the United States hospitals is as follows: On admission the patient is given 8-10 grains of calomel, either in a single dose or in repeated small doses. He is then given at least 4 ounces of cold acetozone solution (made as described above) every two hours. The stock bottle is kept in the refrigerator. At least two pints should be taken in twenty-four hours, and the solution may be used to replace water or other drink. A gentle laxative is occasionally administered, but otherwise no drugs are given. This treatment is said to affect markedly the length and severity of the attacks. The stools become less offensive, and tympanites subsides in two or three days; the colour and consistency of the stools are, however, unaffected. Relapses and hyperpyrexia are said to be unusual under this treatment, and the general condition of the patients is good.

Other diarrhœal conditions may be treated on similar lines. In dysentery and colitis rectal injections of 1 in 1,000 acetozone solution may be employed daily in addition to its administration *per os*. In children suffering from diarrhœal diseases, acetozone may be given as in adults. The solution, however, should be diluted with an equal volume of water and flavoured with orange or lemon syrup. Teaspoonful doses may be given every half-hour at first, the intervals being gradually lengthened as the case improves. Irrigation of stomach and colon with 1 in 3,500 acetozone may also be employed. For the stomach, the irrigations should take place before feeding, and the water used should be warm.

In infective conditions of the eyes a solution of one grain in two fluid ounces is sufficiently strong. This causes a little smarting for a few seconds, but no real damage; a drop or two may be instilled every hour.

For throat work a nebuliser may be employed, or the throat may be swabbed with a 1 per cent. solution every few hours. In simple tonsillitis the results are said to be excellent.

In tinea tonsurans a strong aqueous solution (twenty grains to one quart) may be applied to the scalp. This may apparently cause some soreness of the skin, but no serious inconvenience will follow.

In urethritis a saturated solution (the " stock solution ") may be employed, but this has in some cases given rise to irritation of the mucous membrane, so that it seems advisable to begin with a solution of one half or one third that strength.

As a dressing for suppurating or gangrenous wounds, 2½ grains of acetozone in 4 ounces of water are recommended.

